# *Salvia miltiorrhiza* Extract Prevents the Occurrence of Early Atherosclerosis in Apoe ^-/-^ Mice *via* TLR4/ NF-kB Pathway

**DOI:** 10.2174/1871525721666230206112134

**Published:** 2023-04-25

**Authors:** Ruoyu Wu, Linqi Zhang, Hongjun Xu, Hongxu Chen, Wei Zhao, Yongjie Zhou, Luyang Zhou, Jiangli Wu, Shengjun An

**Affiliations:** 1 Hebei Provincial Engineering Laboratory of Plant Bioreactor Preparation Technology, Hebei University of Chinese Medicine, Shijiazhuang, 050200, China;; 2 Scientific Research Center, Hebei University of Chinese Medicine, Shijiazhuang, 050200, China

**Keywords:** *Salvia miltiorrhiza*,aqueous extract, TLR4/ NF-κB pathway, anti-inflammation atherosclerosis, ApoE^-/-^ mice, coronary arteries

## Abstract

***Objective*:** *Salvia miltiorrhiza* (SM) contains four major aqueous active ingredients, which have been isolated, purified and identified as danshensu (DSS), salvianolic acid A (Sal-A), salvianolic acid B (Sal-B) and protocatechuic aldehyde (PAL), A mixture of these four ingredients is called SABP. Although aqueous extract from *Salvia Miltiorrhiza* has been traditionally used to treat cardiovascular diseases, the efficacy and function of the optimal ratio of SABP in preventing and treating cardiovascular diseases remain unknown. This study aims to explore the anti-inflammatory mechanisms underlying the attenuation of atherosclerosis development by aqueous extract from *Salvia miltiorrhiza*.

***Methods*:** Male ApoE^-/-^ mice (6 weeks) were randomly allocated into three groups: the model group (Model), the SABP group (SABP), and the rosuvastatin calcium group (RC). Male C57BL/6 mice (6 weeks) were used as a control group. All mice were fed with an ordinary diet. After 8 weeks of treatment, the lipid profiles in serum and the lactate dehydrogenase (LDH) and creatine kinase (CK) in heart tissue were measured using an automatic biochemical analyzer. Alterations of the thoracic aorta and the heart were assessed using Hematoxylin and eosin staining. The protein expression of Toll-like receptor 4 (TLR4), TGF beta-activated kinase 1 (TAK1), nuclear factor kappa-B (NF-κB), interleukin-6 (IL-6), and tumor necrosis factor-α (TNF-α) in the heart tissue were determined though immunohistochemistry and western blotting analysis.

***Results*:** The serum low-density lipoprotein cholesterol (LDL-C), triglyceride (TG), and total cholesterol (TC) levels were increased, and the high-density lipoprotein cholesterol (HDL-C) level was decreased in ApoE^-/-^ mice. SABP significantly decreased serum lipid levels and improved histopathology in the thoracic aorta. In addition. SABP treatment inhibited the expression of TLR4, TAK1, NF-κB, IL-6 and TNF-α in the heart in ApoE^-/-^ mice. The LDH and CK in the heart did not differ significantly among different groups, and the heart did not have obvious pathological changes.

***Conclusion*:** These findings indicated that SABP may exert an anti-atherosclerotic effect by lowering blood lipids and inhibiting inflammatory response *via* TLR4/ NF-κB signaling pathway.

## 1 INTRODUCTION

Atherosclerosis (AS) may induce fatal cardiovascular conditions involving the aorta and coronary arteries. Although several mechanisms such as thrombogenic theory, lipid infiltration theory, oxidative hypothesis, and smooth muscle mutation theory have been proposed to be involved in the pathogenesis of AS, it is now widely accepted that inflammation and immunity play an important role in the pathological processing of AS [1, 2]. Toll-like receptor 4 (TLR4)/nuclear factor kappa-B (NF-κB) signaling pathway is involved in the initiation and subsequent progression of AS by regulating the secretion of inflammatory factors [3-6]. Therefore, regulating the key molecules involved in the inflammatory signaling pathway becomes a new approach to preventing and treating AS. In addition, PPARγ, mitogen-activated protein kinase [7], and Jak/STAT3 are also involved in the anti-AS mechanism [8]. It has been reported that high levels of HDL are protective against the progression and complications of AS [9].

*Salvia miltiorrhiza* (SM) is the root of *Salvia miltiorrhiza* of Labiatae, which function as the “decoction of Four Drugs” documented in the ancient books of traditional Chinese medicine. It has been widely used clinically and remarkably affects cardiovascular and cerebrovascular diseases [10]. Because decoction is the traditional mode of using SM, the active ingredients are able to be dissolved aqueously. Studies on aqueous extracts from the SM have shown many new findings. For example, salvianolic acid A (Sal-A) attenuates aortic aneurysm formation in apolipoprotein E(ApoE)-deficient mice [11]. Salvianolic acid B (Sal-B) exerted anti-liver fibrosis effects [12] and anti-inflammatory properties [13]. Danshensu (DSS) and Sal-A prevent cardiac remodeling in spontaneously hypertensive rats [14, 15]. Protocatechuic aldehyde (PAL) protects the cardiovascular system against inflammation and AS [16]. Chinese herbal medicine compatibility is the predominant form for clinical medication. The effective component combination is the new model of Chinese herbal medicine compatibility. SM has four major aqueous extract components: DSS, Sal-A, Sal-B, and PAL, named SABP [17]. The monomer component of traditional Chinese medicine is a current research focus [18] and maybe a potential alternative medicine from SM.

Our laboratory has found that SABP reduces blood pressure [19]. However, the effect of SABP on AS has not been reported. Previous studies mainly focus on the stenosis of vessel lesion caused by AS. This study investigates whether SABP exerts anti-inflammatory roles in protecting the heart in ApoE^-/-^ mice *via* the TLR4/NF-κB signaling pathway. Our data provided new insights into the mechanism of pathogenesis of inflammation-associated diseases and a mechanistic basis for using SABP as a therapeutic approach against inflammation-associated diseases.

## 2 MATERIALS AND METHODS

### 2.1 Chemicals and Reagents

Injectable solutions of DSS (76822-21-4), Sal-A (96574-01-5), Sal-B (115939-25-8), and PAL (139-85-5) was purchased respectively from Shanghai Fu Life Industry Co. Ltd., China. TLR4 antibody (JL-20594R) was obtained from Shanghai Jianglai Science and Technology Ltd. Antibodies against NF-ΚB p65 (GB11142), IL-6 (GB11117), TAK1 (GB11701), TNF-α (GTX110520) and H3 (GB13102-1) were purchased from Wuhan Seville Technology Co., Ltd., GAPDH antibody (AC033) were purchased from AB clonal company. Sheep anti-rabbit secondary antibody, sheep anti-mouse secondary antibody was purchased from KPL company. The Nuclear and Cytoplasmic Protein Extraction Kit (P0027) was purchased from Beyotime Biotechnology Co., Ltd.

### 2.2 Experimental Animals and Treatment

Thirty male ApoE^-/-^ mice and ten C57BL/6J mice (6 weeks of age, 18-22 g) were purchased from Beijing Vital River Laboratory Animal Technology Co., Ltd. (Certificate number SCXK (Beijing): 2016-0010). Mice were housed in a climate-controlled environment (12 h light-dark cycle at 22°C) and fed with a standard diet and water *ad libitum*. After one week of acclimatization, all ApoE^-/-^ mice (n = 30) were randomly assigned into 3 groups: the ApoE^-/-^ (model) group, the SABP group, the rosuvastatin calcium (RC) group. The C57BL/6 mice were defined as the normal control group. The RC group received daily intraperitoneal (i.p.) injections of RC (0.4 mg/kg/d). The SABP group received daily i.p. injections of SABP (DSS: 5 mg/kg/d, Sal-A: 0.233 mg/kg/d, Sal-B: 10 mg/kg/d, PAL: 17 mg/kg/day) [19] in which uniform and orthogonal design formulas were applied to divide into groups and composition. Drugs were dissolved in normal saline. The ApoE^-/-^ model and C57BL/6 J mice received daily i.p. injections of an equal volume of saline. After 8 weeks of treatment, the mice were anaesthetized with 2% isoflurane. The tissues were harvested, then the thoracic aorta and heart were fixed in 4% formalin for histopathological study and the remaining portion was stored at −80°C for biochemical study. All procedures were approved by the Animal Care and Use Committee of Medical Ethics of the Hebei University of Chinese Medicine.

### 2.3 Measurement of Serum Lipid Profiles

Blood samples from the above 4 groups of mice were collected and then centrifuged at 3500 g for 15 min at 4°C. The supernatants were collected and stored at -80°C until use. The levels of total cholesterol (TC), triglyceride (TG), high-density lipoprotein-cholesterol (HDL-C), and low-density lipoprotein-cholesterol (LDL-C) were measured using an automatic biochemical analyzer (iCubio Biomedical Technology Co., Ltd., Shenzhen, China).

### 2.4 Cardiac Function Index

The hearts and aortas were removed and, soaked in 4% polyformaldehyde. After gradient alcohol dehydration, xylene was transparent; the paraffin was embedded. The tissues were sectioned at a thickness of 6 μm. Hematoxylin and Eosin (H&E) staining were performed to observe the pathological changes in the heart and aortas.

### 2.5 Histopathological Study

The heart and aorta were removed and soaked in 4% polyformaldehyde. After a gradient alcohol dehydration, xylene was transparent, the paraffin was embedded. The tissues were sectioned at a thickness of 6 μm. Hematoxylin and Eosin (H&E) staining were performed to observe the pathological changes in the heart and aorta.

### 2.6 Immunohistochemistry Staining

After deparaffinization of paraffin sections, rehydration was done, and the endogenous peroxidase was blocked by 3% H_2_O_2_. Then,the sections were incubated with normal goat serum at 37°C for 20 min and subsequently incubated with rabbit polyclonal antibodies against TLR4 (1:500), NF-κB (1:200), IL-6 (1:400) and TNF-α (1:500) at 4°C overnight. After being rinsed, the sections were incubated with a horse radish peroxidase (HRP)-labeled secondary goat anti-rabbit antibody for 30 min. Antigens were visualized with a diaminobenzidine (DAB) approach. Immunostained paraffin sections were photographed under a light microscope (Leica, Germany) and a proportion of positive immunostained sections were analyzed using Image-Pro Plus 6.0.

### 2.7 Western Blotting

The heart tissues were homogenized, and the lysates were collected to obtain the total protein using lysis buffer (Beijing Solarbio Science and Technology Co., Ltd., Beijing, China). Nuclear proteins were extracted using a nuclear and cytoplasmic protein extraction kit (Beyotime Biotechnology Co., Ltd., Beijing, China), according to the manufacturer’s protocol. Total protein and nuclear protein were extracted and their concentrations in all samples were quantified with the bicinchoninic acid (BCA) protein assay (Applygen Technologies Inc., Beijing, China). Subsequently, proteins were separated on 10% SDS-PAGE gel and transferred to a PVDF membrane. After blocking in 5% skimmed milk for 2 h, the membranes were incubated with primary antibodies against TLR4 (1:1000), NF-κB (1:1000), IL-6 (1:1000), TAK-1 (1:750), GAPDH (1:10000) and H3 (1:10000). Then the membranes were incubated with an HRP-conjugated secondary antibody followed by ECL detection (Vilber Fusion FX5 Spectra, Paris, France). And the bands were analyzed semi-quantitatively with Image J software (National Institutes of Health, Bethesda, Maryland, USA).

### 2.8 Statistical Analysis

All the data were expressed as mean ± standard deviation error of the mean (SEM). SPSS 19.0 software was used for one-way analysis of variance (ANOVA) and *post hoc* repeated measurement. A value of *P* < 0.05 was considered statistically significant.

## 3 RESULTS

### 3.1 Effect of SABP on Lipid Levels in Serum

**Fig. (1):**
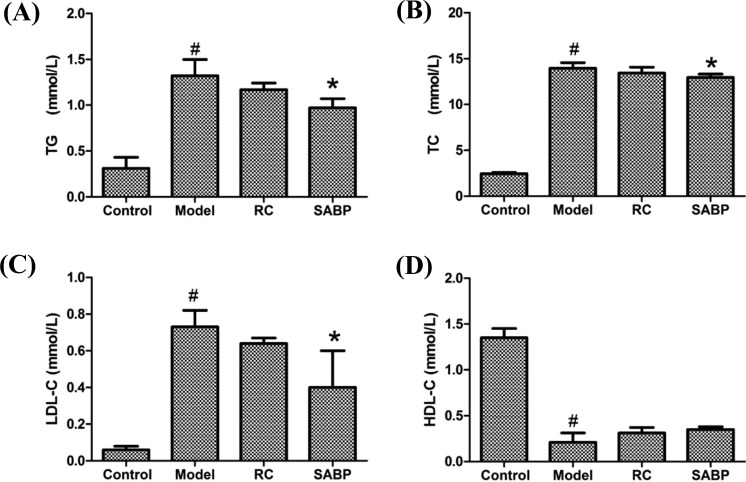
TG, TC, LDL-C and HDL-C serum lipid levels in response to SABP treatment. (**A**) The TG level; (**B**) The TC level; (**C**) The LDL-C level; (**D**) The HDL-C level. Data are presented as the mean ± SD. N = 6 in each group. #*P* < 0.05, compared with control group;*P < 0.05, compared with the model group.

ApoE^-/-^ mice showed higher levels of TC, TG and LDL-C and a lower level of HDL-C in the model group than mice in the control group. SABP treatment decreased the serum TG, TC, and LDL-C levels in ApoE^-/-^ mice (*P* < 0.05) (Fig. **1**). SABP treatment slightly increased HDL-C levels was ApoE^-/-^ mice (*P* > 0.05). However, there were no differences in serum TC, TG, LDL-C and HDL-C levels between the model group and those treated with RC. These data indicated that SABP treatment dramatically altered the serum lipid profile in normal diet-fed ApoE^-/-^ mice.

### 3.2 Effect of SABP on LDH and CK Levels in Heart

**Fig. (2):**
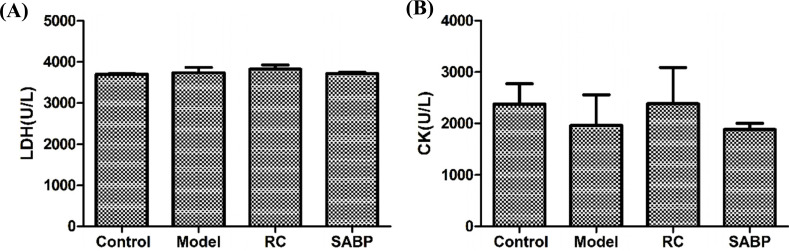
The LDH and CK levels in heart in response to SABP treatment. (**A**) The LDH level in heart; (**B**) The CK level in heart. Data are presented as the mean ± SD. N = 6 rats in each group.

However, we found that the LDH and CK in heart tissues did not differ in control, ApoE^-/-^ mice, SABP-treated ApoE^-/-^ mice (Fig. **2**), suggesting that the cardiac function did not alter in ApoE^-/-^ mice.

### 3.3 The Thoracic Aorta and Cardiac Histological Changes

**Fig. (3):**
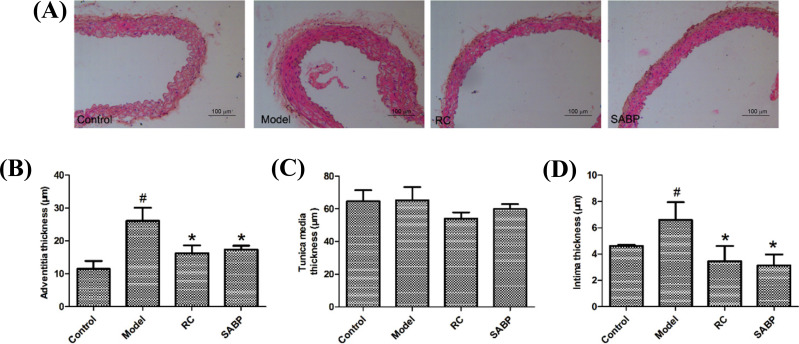
SABP attenuated degree of AS-related lesion of aorta in ApoE^-/-^ mice. (**A**) HE staining of aorta; (**B**) The adventitia thickness; (**C**) The tunica media thickness; (**D**) The intima thickness. Data are presented as the mean±SD. n=6. ^#^*P* < 0.05, compared with control group;*P < 0.05, compared with the model group.

In the control group, the three layers of the aorta were clear; the intimal surface was covered with monolayer flat endothelial cells. The tunica media was composed of smooth muscle cells with the neat arrangement. The outer membrane was intact, and the inner wall of the official cavity was smooth. No abnormal substance proliferation was found. In the model group, the vascular wall structure was disordered and the intima was seriously injured and thickened. The arrangement of smooth muscle cells in the middle layer was disordered. No AS plaques were found in the official cavity. In the RC and SABP group, the vascular endothelial cells had no obvious morphological changes and the smooth muscle cells of the tunica media were arranged neatly (Fig. **3D**). As presented in Figs. (**3A**-**C**), the thickness of both adventitia and intima were increased in the model group compared with the control group, the RC and SABP groups (*P* < 0.05). The tunica media thickness did not have any obvious changes among the groups (*P* > 0.05).

**Fig. (4):**
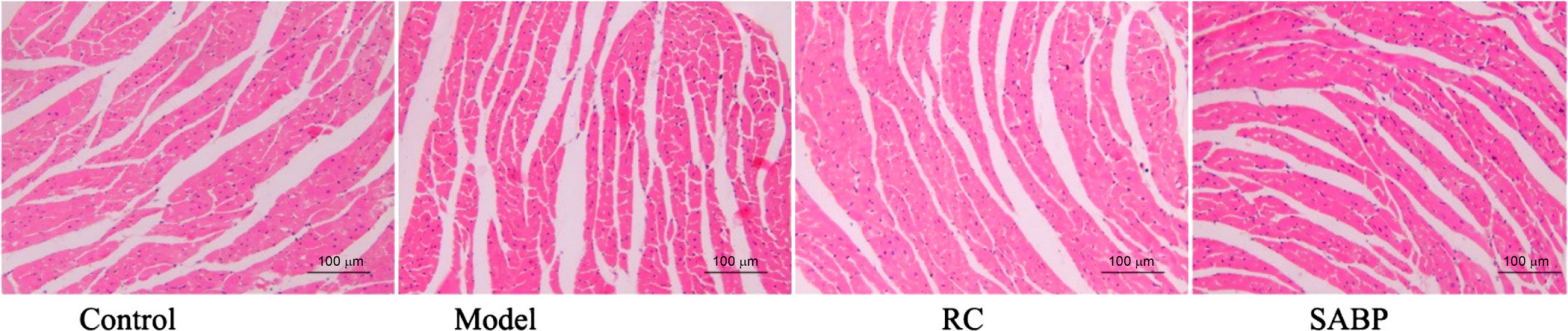
Observation of histologic changes of heart by HE staining. The myocardial fibers in the model and other three groups were arranged neatly, the cell nucleus of cardiac myocyte was located in the center of the cells and shaped in oval or round. No thickening, no congestion, hemorrhage and other lesions were found in the myocardial fibers.

The myocardial fibers in the model and the other three groups were arranged neatly, the cell nucleus of the cardiac myocyte was located in the center of the cells and shaped oval or round. No thickening, no congestion, hemorrhage and other lesions were found in the myocardial fibers (Fig. **4**).

### 3.4 SABP Regulated Expression of the TLR4/NF-κB Signal Pathway in the Heart

**Fig. (5):**
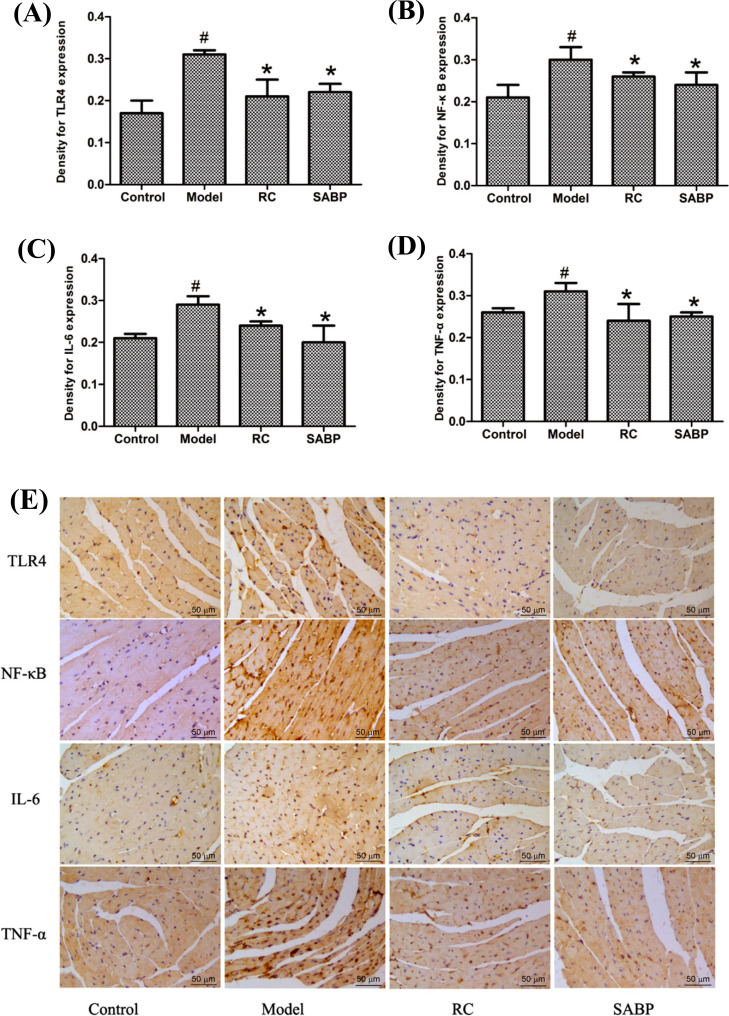
SABP suppressed TLR4, NF-κB, IL-6 and TNF-α expression in the heart. (**A**-**D**) Normalized quantitative data for TLR4, NF-κB, IL-6 and TNF-α protein expression levels; (**E**) The TLR4, NF-κB, IL-6 and TNF-α expression were displayed by immunohistochemistry staining. The arrows showed the staining for targeted protein. Values are expressed as mean ± SD, n = 4. ^#^*P* < 0.05, compared with control group;*P < 0.05, compared with the model group.

**Fig. (6):**
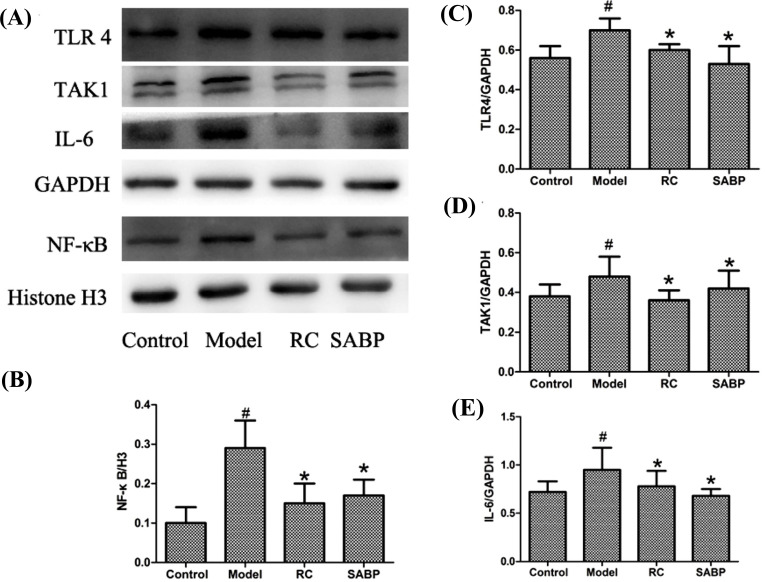
SABP treatment regulated the TLR4, NF-κB, IL-6 and TAK1 expression in the heart. (**A**) Western blotting bands showed the TLR4, TAK1 NF-κB and IL-6 and protein expression levels. (**B**-**E**) Normalized quantitative data for NF-κB, TLR4, TAK1 and IL-6 protein expression. Values are expressed as mean±SEM, n = 4. ^#^*P* < 0.05, compared with control group; *P < 0.05, compared with the model group.

In order to elucidate the molecular mechanism underlying SABP anti-inflammation effect, we evaluated the expression of TLR4, TAK1, activated NF-κB, IL-6, and TNF-α in the heart by immunohistochemistry staining and western blotting analysis. Immunohistochemistry staining and Western blotting analysis indicated that the expression of TLR4, IL-6, TNF-α, and TAK1 proteins were upregulated, and NF-κB was activated in the heart tissue of ApoE^-/-^ mice in the model group (Figs. **5** and **6**). Compared with ApoE^-/-^ mice in the model group, TLR4, TAK1, IL-6, and TNF-α protein were significantly decreased, and activated NF-κB was inhibited in the SABP group (*P* < 0.05). These data indicated that SABP treatment regulated the AS by affecting the TLR4/NF-κB pathway and associated proteins.

## 4 DISCUSSION

This study demonstrated that SABP, a mixture of aqueous extract of SM, has an efficient anti-atherosclerotic effect in all dose ratios of four aqueous components by uniform and orthogonal designated formulas. *Salvia miltiorrhiza* is a topical Chinese herbal medicine (CHM) for improving cardiovascular function. Studies have shown that the active ingredients of SM are hydrophilic phenolic acids. SABP are the effective aqueous extract components of SM and it is the new model of Chinese herbal medicine compatibility. This mixture of the four monomers might contribute to anti-atherosclerotic effects of the medicinal herbs.

We found that AS was formed initially in ApoE^-/-^ mice fed with a common diet for 8 weeks. Expressions of inflammatory factors and key proteins in the TLR4/NF-κB signal pathway were increased in the ApoE^-/-^ mice group compared with the C57BL/6 mice group. The data presented here suggested that SABP treatment could attenuate AS by reducing the inflammatory response.

ApoE^-/-^ mice could form hyperlipidemia and other form plaques, regardless of whether they were fed a basic or high-fat diet. In this study, after 8 weeks of feeding with a common diet, serum TC, TG and LDL-C in the model group were significantly increased and HDL-C was significantly decreased, indicating that ApoE^-/-^ mice had serious hyperlipidemia. However, no plaques were found in the HE staining of the thoracic aorta. Likely, 8-week normal diet feeding in 6-week-old ApoE^-/-^ mice were not enough to form lipid plaque. Studies have reported that AS plaque was formed after 6-week-old ApoE^-/-^ mice feeding with a high-fat diet for 13 weeks [20]. Additionally, the cardiac function indexes and the pathology results indicated that ApoE^-/-^ mice heart did not have obvious pathological changes. Our finding is consistent with a previous Tian *et al.* [21] study showing that cardiac function indexes were not altered in 12-month-old ApoE^-/-^ atherosclerotic mice.

Statins are widely used in the prevention and treatment of AS because of their protective effects on vascular endothelial cells, anti-inflammation and lipid lowering [22, 23]. However, it has been shown that statins are not able to reduce cholesterol in ApoE^-/-^ mice because of the lack of critical ligands for LDL receptor (ApoE), which is important for lowing cholesterol [23, 24]. This notion has been supported by studies done by Song Ke and Yang [25, 26]. This study's results are consistent with previous studies' findings that statins cannot reduce the level of blood lipids in ApoE^-/-^ mice. However, whether SABP has effects on serum levels of lipids is still not clear. In this study, we demonstrated that SABP treatment lowered TC, TG and LDL-C. Although lipid metabolism is important and linked to AS, reducing blood lipids alone does not account for the mechanism underlying the ability of SABP in the prevention and treatment of AS.

The TLR4 signal pathway associated with inflammation has been discovered in many conditions, such as colitis [27], and intracerebral hemorrhage [28]. Growing evidence suggests that the TLR4 signal pathway might be involved in inflammation during AS [29, 30]. The TLR4-triggered intracellular signal pathway recruits downstream proteins and activates NF-κB, which leads to the release of NF-κB and nuclear translocation, followed by upregulating the expression of inflammatory mediators including IL-6 and TNF-α [30-32]. Our results revealed that SABP reduced the expression of TLR4 and TAK1. Thus, we hypothesized that the anti-inflammation mechanism of SABP is related to TLR4 signals. We also examined the protein levels of NF-κB in the nucleus and found that SABP inhibited the activation of NF-κB. We further examined the expression of inflammatory factors IL-6 and TNF-α. We found that SABP could profoundly inhibit the expression of these inflammation-related molecules. These findings suggest that SABP suppresses the AS by virtue of inhibition of the TLR4/NF-κB signaling pathway, accompanied by repression of inflammatory factors.

For the first time, we demonstrated that SABP played an important role in attenuating AS by downregulating the key protein expression in the TLR4/NF-κB pathway. Our present study revealed the mechanism that SABP inhibited AS-related inflammation, partly through inhibition of the TLR4/NF-κB activity. If further study is needed, it is necessary to block this pathway in the subsequent *in vitro* experiments to identify the specific components in the signaling pathways involved in the effect of SABP.

## CONCLUSION

In conclusion, our study is the first to demonstrate that SABP alleviated the development of AS. The underlying mechanism of SABP was associated with improving blood lipids metabolism and inhibiting the TLR4/NF-κB signaling pathway. These results indicate that the efficacy and function from the optimal compatibility ratio of SM active ingredients (SABP) represents a new strategy to develop the anti-atherosclerotic effect produced by SM.

## Data Availability

The authors confirm that the data used to support the findings of this study are included in the article.

## LIST OF ABBREVIATIONS

ANOVA: Analysis of Variance
AS: Atherosclerosis
BCA: Bicinchoninic Acid
H&E: Hematoxylin and Eosin
HRP: Horse Radish Peroxidase
PAL: Protocatechuic Aldehyde
RC: Rosuvastatin Calcium
Sal-A: Salvianolic Acid A
Sal-B: Salvianolic Acid B
SM: *Salvia miltiorrhiza*
TC: Total Cholesterol
TG: Triglyceride
